# Reactive oxygen species a double-edged sword for mesothelioma

**DOI:** 10.18632/oncotarget.4253

**Published:** 2015-06-10

**Authors:** Serena Benedetti, Barbara Nuvoli, Simona Catalani, Rossella Galati

**Affiliations:** ^1^ Department of Biomolecular Sciences, University of Urbino “Carlo Bo”, Urbino, Italy; ^2^ Molecular Medicine Area, Regina Elena National Cancer Institute, Rome, Italy

**Keywords:** reactive oxygen species, mesothelioma, cell trasformation, cell proliferation, therapy

## Abstract

It is well known that oxidative stress can lead to chronic inflammation which, in turn, could mediate most chronic diseases including cancer. Oxidants have been implicated in the activity of crocidolite and amosite, the most powerful types of asbestos associated to the occurrence of mesothelioma. Currently rates of mesothelioma are rising and estimates indicate that the incidence of mesothelioma will peak within the next 10–15 years in the western world, while in Japan the peak is predicted not to occur until 40 years from now. Although the use of asbestos has been banned in many countries around the world, production of and the potentially hazardous exposure to asbestos is still present with locally high incidences of mesothelioma. Today a new man-made material, carbon nanotubes, has arisen as a concern; carbon nanotubes may display ‘asbestos-like’ pathogenicity with mesothelioma induction potential. Carbon nanotubes resulted in the greatest reactive oxygen species generation. How oxidative stress activates inflammatory pathways leading to the transformation of a normal cell to a tumor cell, to tumor cell survival, proliferation, invasion, angiogenesis, chemoresistance, and radioresistance, is the aim of this review.

## INTRODUCTION

Oxidative stress is defined as an imbalance between the production of free radicals and reactive metabolites, so-called oxidants or reactive oxygen species (ROS), and the ability of a biological system, named antioxidant, to readily detoxify reactive intermediates or repair the resulting damage [1]. ROS are constantly generated under normal conditions as a consequence of aerobic metabolism. The most common ROS types such as superoxide anions (O_2_^−^), hydrogen peroxide (H_2_O_2_), and hydroxyl radicals (HO·) are produced by biological reduction of molecular oxygen [2]. The electron transfer to molecular oxygen occurs at the level of the mitochondrial respiratory chain [3, 4]. Under hypoxic conditions, the mitochondrial respiratory chain also produces nitric oxide (NO), which can generate reactive nitrogen species (RNS) [5].

**Figure 1:**
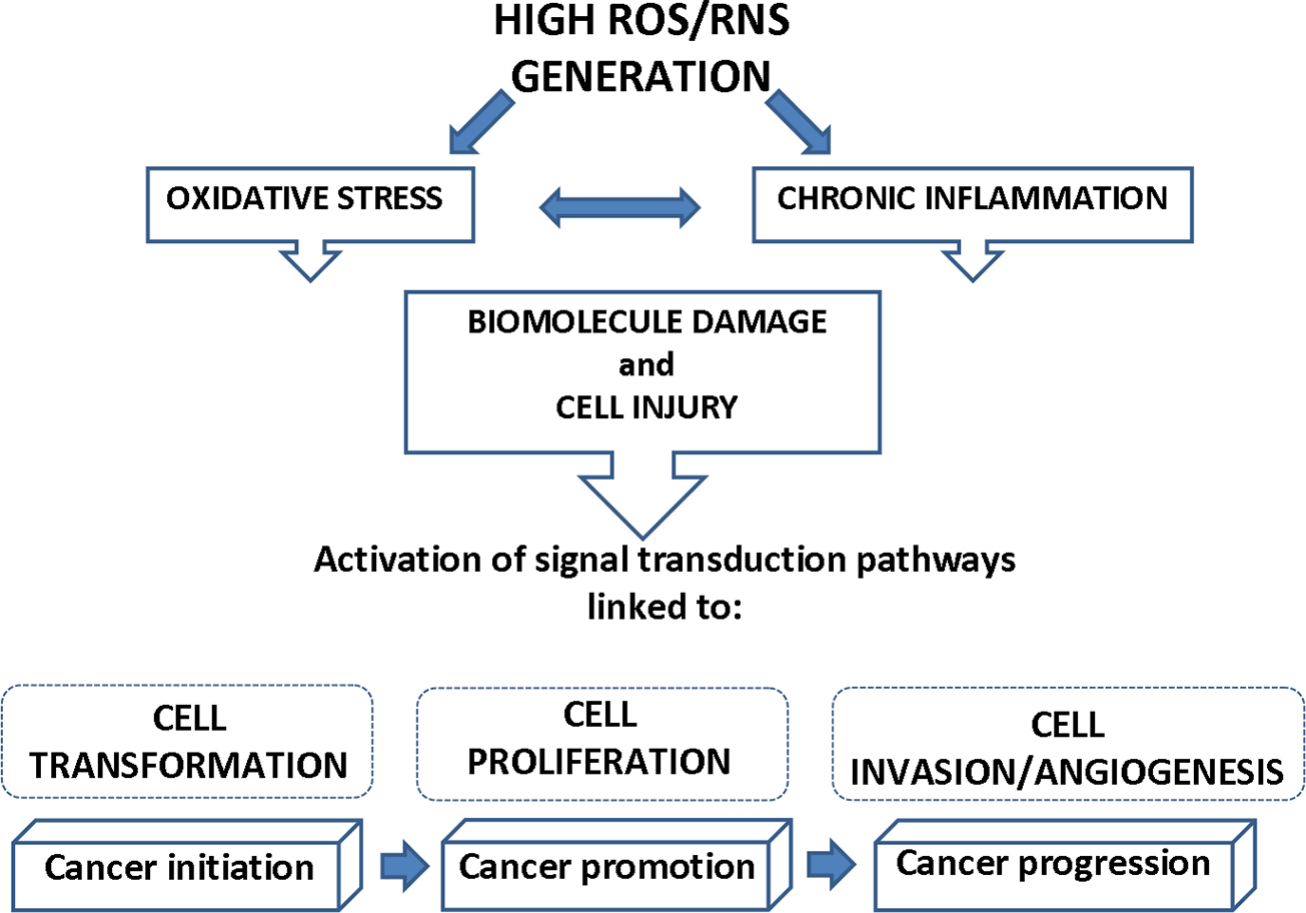
Link between ROS/RNS generation and cancer High concentration of ROS and RNS, leading to oxidative stress and chronic inflammation, can cause macromolecule damage and cell injury that, in turn, activate signal transduction pathways linked to the progressive phases of carcinogenesis (cell transformation, cell proliferation, cell invasion and angiogenesis).

ROS can react with DNA, proteins, carbohydrates, and lipids in a destructive manner due to their high levels of chemical reactivity. Thus, ROS are considered DNA-damaging agents that increase mutation rates and promote oncogenic transformation, and act as cellular messengers in redox signaling causing disruptions in normal mechanisms of cellular signaling [6, 7] (Figure 1). Enzymatic (i.e. superoxide dismutase, catalase, and glutathione peroxidase) and nonenzymatic (i.e. glutathione) antioxidants normally counteract damaging effects of intracellular ROS by either repairing the oxidative damage or directly scavenging oxygen radicals.

In humans, oxidative stress is thought to be involved in the development of cancer [8], and in a wide spectrum of diseases, including chronic inflammation [8–10] (Figure 1). Inflammation is part of the complex biological response to harmful stimuli, such as pathogens, allergens and toxic chemicals. An acute inflammatory response is usually beneficial, and can also be anti-tumorigenic and have a role in tumor suppression [11]. In some disorders, the inflammatory process, which under normal conditions is self-limiting, becomes continuous, and chronic inflammatory diseases might develop subsequently [12]. Epidemiological evidence suggests that 1 out of 4 of all cancers are due to chronic infection or other types of chronic inflammation [13–16]. Chronic inflammation predisposes cells to oncogenic transformation by a variety of mechanisms, including the induction of genomic instability, increasing angiogenesis, altering the genomic epigenetic state, and increasing cell proliferation [17]. Overproduction of reactive oxygen and ROS, aberrant inflammatory cytokine and chemokine expression, increased cyclooxygenase-2 (COX-2), and nuclear factor kappa B (NFkB) expression are just some of the molecular factors that contribute to inflammation-induced carcinogenesis [18]. For example, chronic inflammatory bowel disease is a predisposing factor of colon cancer, chronic B and C hepatitis are predisposing factors of hepatocellular carcinoma, and chronic gastritis induced by *Helicobacter pylori* is a predisposing factor of gastric cancer [19–21]. Similarly, there are studies investigating the link between chronic inflammation associated with long-term asbestos exposure and mesothelioma [22, 23]. Chronic inflammation triggered by asbestos exposure leads to increased production of ROS from inflammatory cells, or alteration of immunocompetent cells and later reduction of tumor immunity [24, 25]. Free radicals generated from asbestos fibers and/or damages by fibers can alter biological macromolecules including proteins, cell membrane lipids, deoxyribonucleic acid (DNA), and ribonucleic acid (RNA) resulting in the initiation of numerous signal transduction pathways that are linked to inflammation, malignant transformation, proliferation, and apoptosis. How oxidative stress modulates these cellular processes in mesothelioma is the focus of this review, after considering the generation of ROS by asbestos.

### Asbestos and oxidants in mesothelioma development

Malignant mesothelioma (MM) is a tumor arising from mesothelial cells after asbestos exposure.

Asbestos fibers are naturally occurring in rocks and soils, and consist of six distinct types. The amphibole types of asbestos (crocidolite, amosite, anthophyllite, tremolite, and actinolite) are rod-like and more durable in the body than the only serpentine asbestos type, chrysotile [26]. The hazard posed by fibres relates to the mesothelial lining of the pleural cavity and to some extent the peritoneal cavity. Individuals exposed to asbestos demonstrate a wide range of pleural pathologies including pleural effusion (a build up of fluid within the pleural space), pleural fibrosis and pleural mesothelioma [27]. A variable, usually small, proportion of mesotheliomas developing in individuals exposed to asbestos arise in the peritoneal cavity, likely as a result of fibre translocation from the pleural cavity to the peritoneal cavity [28]. It has been postulated that the toxicity of fibres is related to fibre length, bio-persistence, and dose; a hypothesis known as the ‘fibre paradigm’ [29].

**Figure 2:**
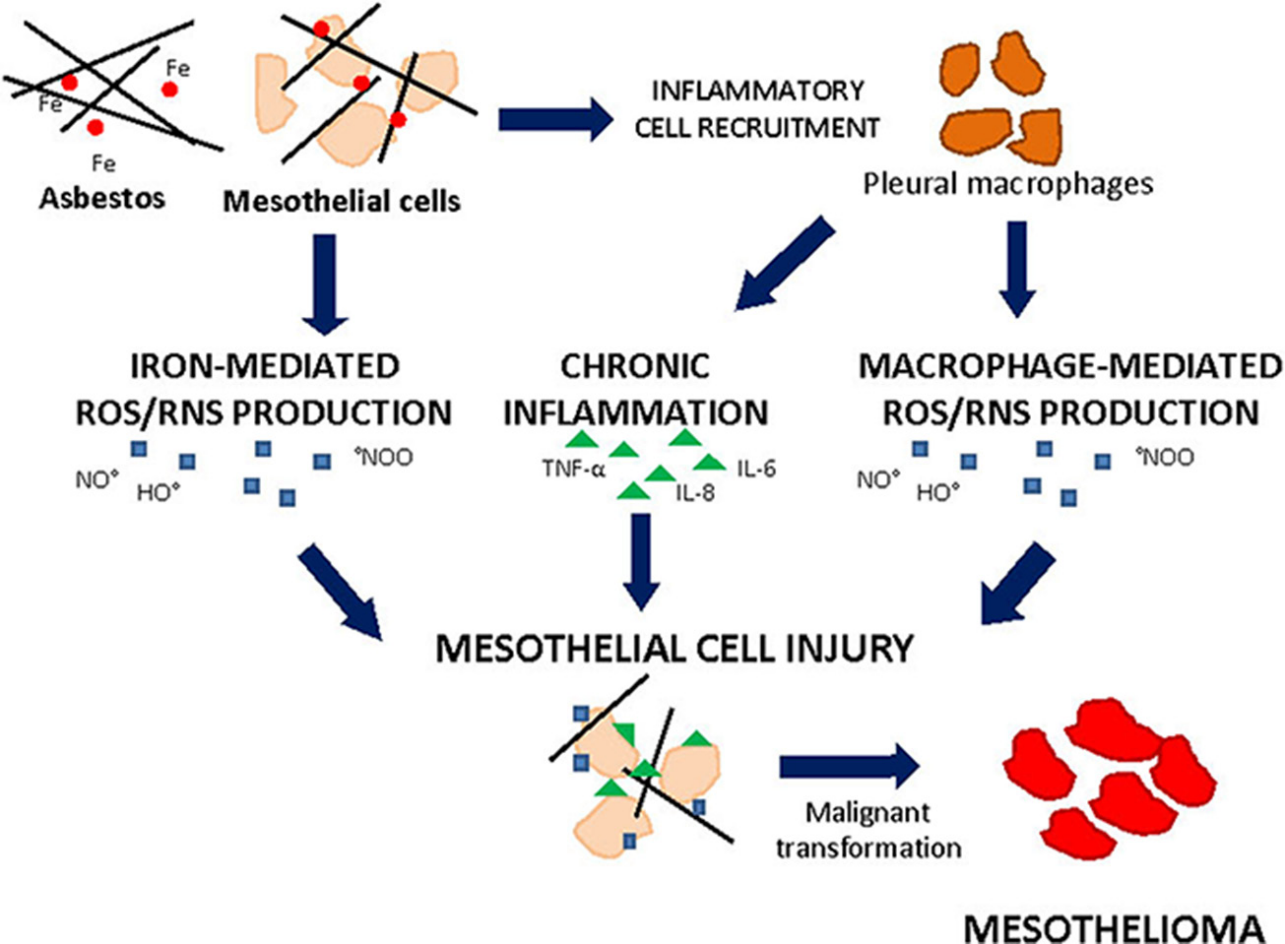
Asbestos-induced cell injury leading to mesothelioma Mesothelial cells exposed to iron- and macrophage-mediated ROS/RNS production and inflammatory cytokines can cope cell injury and undergo malignant transformation giving rise to mesothelioma.

Fibre dimension is important in determining the respirability of the material and its deposition in the respiratory tract. It has been shown that fibre length is also a critical parameter determining its fate *in vivo* [29]. Indeed, above a certain length, a fibre may not be readily engulfed by cells from the immune system. The retention of long fibres at the stomatal openings on the parietal pleura, coupled with frustrated phagocytosis of pleural leukocytes that attempt to ingest them, produce an oxidative stress and a chronic pleural mesothelial inflammatory response which may result in disease [29]. Mesothelial cells internalize the fibers via integrins or other receptors; fibre uptake was found in some studies to be necessary for adverse effects of the fibers such as ROS generation, DNA damage, and apoptosis [30]. Asbestos produces ROS by at least two principal (Figure 2). The first mechanism involves the iron content of the fibre augmenting HO. formation through iron-catalysed reactions. The second mechanism implicates the release of ROS upon activation of inflammatory cells. Asbestos also generates RNS such as nitric oxide (·NO) and peroxynitrite (·ONOO) [31]. It has been demonstrated that H_2_O_2_, O_2_^.−^ and RNS are released from several types of asbestos fibers in cell-free solutions or in cells, especially alveolar or peritoneal macrophages, after phagocytosis of asbestos fibers *in vitro* or after inhalation [32]. Uptake of asbestos fibers, the leading cause of mesothelioma, results in accumulation of ROS and RNS which act as second messengers of asbestos-mediated carcinogenesis.

### Generation of ROS by asbestos

All types of asbestos have iron cations as an integral component of the crystalline structure, as a substitute cation, or as a surface impurity [33]. The high iron content of asbestos types appears to be critical to the genesis of ROS. The iron associated with asbestos promotes the formation of the highly reactive HO. from H_2_O_2_ with oxidation of ferrous iron (Fe^2+^) to ferric iron (Fe^3+^) (Fenton reaction) [33]. In addition, asbestos can act as a catalyst for the generation of ROS by an iron-mediated Haber-Weiss reaction [34]. These highly chemically reactive molecules modify DNA (particularly mitochondrial and telomeric DNA), proteins (including DNA repair enzymes), and lipids. Asbestos-induced cell damage by iron-catalyzed formation of ROS [31, 35], involves DNA strand breaks [36] and oxidant-induced base modifications. 8-Hydroxy-2′-deoxyguanosine (8OHdG), a major product of such oxidative damage [37], causes G→T and A→C transversions [38]. These substitutions have been reported as the sites of spontaneous oncogene expression and may be largely responsible for the onset of carcinogenesis and cell proliferation, ultimately leading to cancer manifestation [37–40]. Most of the necessary mutations occur early during cancer development, also resulting in processes such as chronic inflammation, together providing the environment to expand and select malignant clones.

High levels of G→T transversions in DNA of the omenta (a part of peritoneum), a relevant target tissue for mesothelioma carcinogenesis, were found in rats treated with asbestos [40]. 8OHdG levels have been analysed in the peripheral blood cells of asbestos-exposed workers and MM patients and compared with those of age-matched healthy controls [41]. Human exposure to asbestos fibers was found to increase significantly the steady state content of 8OHdG in lymphocyte DNA of asbestos-exposed workers [42]. In addition, individuals who had been exposed to asbestos fibres showed two to four times more DNA double-strand breaks in white blood cells than non-exposed persons [43]. DNA repair mechanisms play a key role in limiting the extent of DNA damage and the accumulation of damaged DNA bases. Although a significant delay in DNA repair was found in MM patients, no difference in DNA repair rate was observed between asbestos-exposed subjects and unexposed controls [44].

ROS may attack biological macromolecules such as membrane lipids and lead to their peroxidation. Great diversity of aldehydes are formed when lipid hydroperoxides break down in biological systems. Some of these aldehydes are highly reactive and may be considered as second toxic messengers which disseminate in cells and tissues and produce additional damage, including additional lipid peroxidation, oxidative stress, and oxidative attack to DNA [45–47]. Plasma malondialdehyde (MDA) (an indicator of lipid peroxidation) was determined in 97 randomly selected asbestos-exposed workers and in 42 healthy male controls. MDA in asbestos-exposed workers was significantly higher than in controls. Neither age nor smoking was related to MDA levels both in controls and exposed workers [48].

### Generation of RNS by asbestos

Asbestos has been shown to induce the expression and activity of constitutive or inducible nitric oxide synthase (iNOS) in alveolar macrophages and mesothelial cells [49]. iNOS produces enzymatically from arginine nitric oxide (•NO) which can interact with O_2_• to form peroxynitrite (•ONOO), a highly reactive oxidant that attacks a variety of biological targets [50, 51] and that may form •HO as free radical by an iron-independent mechanism [50, 51]. Interestingly, •NO also attenuates H_2_O_2_-induced lipid peroxidation and pulmonary artery endothelial cell injury, suggesting that it has antioxidant functions [51, 52].

Using an *in vitro* luciferase model, it was demonstrated that crocidolite activates the iNOS promoter. Moreover, increased steady state levels of iNOS mRNA and production of •NO/•ONOO by alveolar macrophages isolated from rats were seen after inhalation of asbestos. This effect was reduced by the NOS inhibitor, NG-monomethyl-L-arginine [53]. Additionally, strong immunoreactivity for nitrotyrosine, a marker of •ONOO formation, was detected in the lung and pleural mesothelium from chrysotile- and crocidolite-exposed rats [54]. The majority of malignant mesotheliomas express strong iNOS immunoreactivity. In contrast, its expression is infrequently found in non-neoplastic healthy mesothelium [55].

### Oxidative stress and mesothelio inflammation

Asbestos exposure is known to increase the risk of pulmonary pathologies in the form of non-malignant inflammatory diseases, such as pleural plaques, pleural effusions and asbestosis, and malignant diseases, such as mesothelioma and bronchogenic carcinoma. Pleural plaques are produced by the effect of recurrent inflammatory and repair processes occurring for long time periods. Chronic inflammatory episodes may predispose to malignant evolution, as it is known that the majority of MM develops on pleura affected by pleural plaques and not on the normal pleura [56]. Various mechanisms have been advanced for the pathogenesis of asbestos-induced mesothelioma [57]. One proposed mechanism is the oxidative stress concept that highlights how iron within asbestos fibers catalyzes free radical generation and thereby induces oxidative stress and carcinogenesis [32]. Another proposed mechanism is the chronic inflammation by asbestos. Fibre-induced inflammation in the parenchyma reverses both the normal flow of lymph and the normal transpleural pressure, resulting in a net flow of fluid and fibers directly into the pleural space from the underlying parenchyma [58]. This leads to mesothelial and endothelial cell damage, inflammation and accumulation of pleural macrophages. Pleural macrophages undergo frustrated phagocytosis in an attempt to enclose the long fibers. Over time, inflammation becomes chronic and plays an important role in asbestos-induced carcinogenesis that is characterized by persistent release of cytokines and oxidants from macrophages that ultimately lead to further inflammation, fibrosis and genotoxicity in bystander mesothelial cells. Increased pathogenicity of long asbestos fibers depends on the persistent presence of fibers, repeated fibre-induced injury, tissue repair and local inflammation [59]. The mouse peritoneal cavity has been used as a model of direct mesothelial exposure, and much greater inflammatory responses were evidenced in mice exposed to high doses of long fibres than in those exposed to shorter fibres. *In vitro* systems have also demonstrated the greater potency of long compared to short fibres in assays of pro-inflammatory and genotoxic activity [60]. Many cytokines and growth factors are shown to be implicated in asbestos-induced MM pathogenesis, including Tumor Necrosis Factor alpha (TNF-α), Transforming Growth Factor Beta (TGF-β), Platelet-Derived Growth Factor (PDGF), Insulin-like Growth Factor (IGF), interleukin-6 (IL-6), interleukin-8 (IL-8), Vascular Endothelial Growth Factor (VEGF), and Hepatocyte Growth Factor (HGF) [56]. TNF-α is released in response to large accumulations of macrophages undergoing phagocytosis of asbestos. The binding of the released TNF-α to its receptor, TNF-R1, which is also expressed by mesothelial cells and activated by NFκB pathway, increases the percentage of human mesothelial cells that survive to asbestos exposure [61]. In addition, asbestos exposure causes increased inflammatory responses that include IL-1β, IL-13, basic Fibroblast Growth Factor (bFGF), VEGF, and granulocyte colony stimulating factor (G-CSF) release, which may be responsible for mesothelioma transformation of these cells [62].

### ROS and mesothelioma cell transformation

Cancer is a multistage process defined by at least three stages: initiation, promotion, and progression. Initiation is defined as a change in genetic material, manifested by DNA damage, mutations, or other DNA heritable changes. These genetic alterations may be rendered by increased expression of oncogenes or decreased expression or function of tumor suppressor genes. Initiation alone does not render a cell tumorigenic. Additional signals in “tumor promotion” are required for the expansion of the initiated cell population and subsequent genetic changes. A hallmark of the “tumor promotion” is the modulation of gene expression resulting in increased cell number either through cell division and/or decrease in apoptotic cell death [63]. Following additional chemical insults or through multiple divisions and acquisition of mutations in the preneoplastic focal lesions, the formation of benign and/or malignant neoplasms can occur during the progression stage. Genetic predisposition may play a role in the susceptibility of individuals to certain carcinogens as suggested for mesothelioma found in certain regions of Turkey [64].

Asbestos-induced free radical production results from both direct (e.g. fibre) and indirect (e.g. inflammatory cell recruitment) mechanisms. ROS/RNS elaborated by asbestos may also play a role in tumor promotion, either by stimulating initial mesothelial or epithelial cell damage and subsequent compensatory hyperplasia, or by altering the cell cycle kinetics of initiated mesothelial or epithelial cells [65]. ROS are involved in the link between chronic inflammation and cancer [21]. Indeed, an important characteristic of tumor promoters is their ability to recruit inflammatory cells and to stimulate them to generate ROS [29]. Asbestos and its second messengers, ROS and RNS, cause mutations, altered DNA bases, DNA single-strand, break chromosomal alterations and sister chromatid exchange. Asbestos-induced DNA base pair alterations are probably caused by •HO and .ONOO since these oxidants commonly react with DNA to produce hydroxylated bases or DNA single-strand [66, 67]. As reviewed elsewhere, asbestos promotes 8-OHdG formation in DNA in cell free systems [37, 38]. Iron-catalysed free radicals derived from peroxides or organic hydroperoxides can also augment asbestos-induced DNA damage in cell-free systems [68]. Asbestos-induced DNA damage by iron-derived •HO can also occur in relevant target cells [69]. A complex profile of somatic genetic changes has been revealed in human MM. These changes implicate a multistep process of tumorigenesis. The occurrence of multiple, recurrent cytogenetic deletions suggests that loss or inactivation of tumor suppressor genes is critical to the development and progression of mesothelioma. Deletions of specific regions in the short (p) arms of chromosomes 1, 3, and 9 and long (q) arms of 6, 13, 15, and 22 are repeatedly observed, and loss of a copy of chromosome 22 is the single most consistent numerical change [70]. Relatively little is known about the early changes in the genesis of mesothelioma. Among the known cytogenetic changes, the most frequent is the loss of p16/CDKN2A-p14ARF at 9p21(by homozygous deletion) [71], adversely affecting both Rb and p53 pathways, respectively. NF2 (merlin), a tumor suppressor located at 22q12, is also frequently altered in mesotheliomas (by an inactivating mutation coupled with allelic loss) [72–73].

### ROS and mesothelioma cell survival

**Figure 3:**
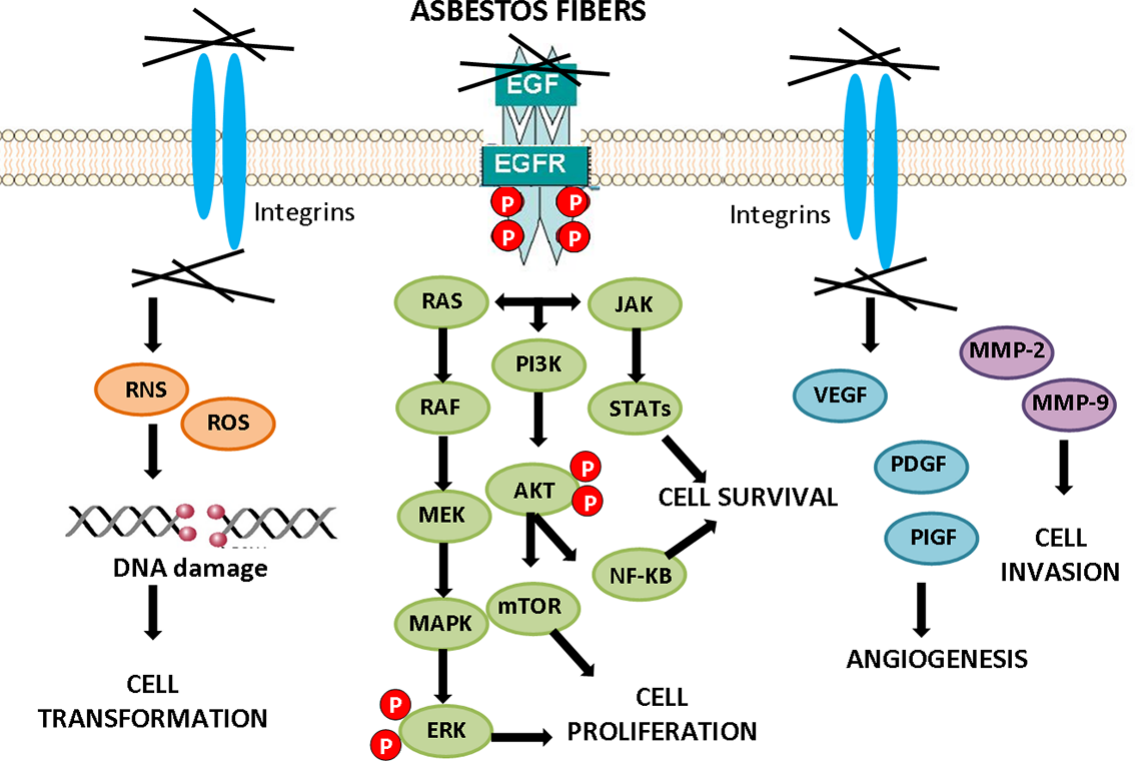
Cell signaling activation by asbestos Exposure to asbestos fibers leads to the activation of downstream signaling cascades conducing to cell transformation, cancer cell survival and proliferation, angiogenesis and invasion.

One of the key characteristics of tumor cells is their increased ability to survive compared with normal cells. Survival pathways may be activated by direct interaction of asbestos fibers with receptors on the cell surface and by interaction with integrins, or via elaboration of ROS, and are often up-regulated in MMs, in which they contribute to tumor development, homeostasis, and resistance to chemotherapy [74] (Figure 3).

Epidermal growth factor receptor (EGFR)-linked survival pathways including extracellular signal-regulated kinases (ERKs) 1/2 [75–77], phosphoinositol-3-kinase (PI3K)/protein kinase B (AKT) signaling pathways [78], and the downstream mTOR are involved in cell growth and survival, and they are often found to be activated in mesothelioma [79]. The activation of the EGFR by asbestos fibers and instigation of these survival cascades may allow a population of asbestos-altered mesothelial cells to be selected and/or expanded in a potentially adverse environment, such as that associated with oxidant-generating asbestos fibers.

The PI3K/Akt signaling pathway is the best-characterized pathway in cell survival and its activity can be redox-regulated [80]. Being the signal transduced via phosphorylation, phosphatases are the most important negative regulators. One such phosphatase is the lipid phosphatase PTEN (Phosphatase and Tensin homologue), which can catalyze the opposite reaction of PI3-kinase. PTEN, like other phosphatases, requires reduced cysteines in its active site for activity. Hydrogen peroxide can reversibly inhibit PTEN by oxidation of these key cysteines, resulting in the activation of the PI3-kinase/Akt pathway [81]. Activation of the mammalian target of rapamycin (mTOR) signalling contributes to the pathogenesis of many tumor types [82]. Akt directly phosphorylates and activates mTOR [83]. Akt is also an inhibitor of apoptosis because of its ability to inactivate proapoptotic molecules, including caspase-9 and the Bcl-2 homology domain 3-only protein Bcl-XL/Bcl-2-associated death promoter, and by triggering the activity of the transcription factor NF-κB. AKT is frequently activated in MM cells. Malignant mesothelioma tumor specimens demonstrate high levels of phosphorylated Akt expression; in addition, a poor survival of malignant pleural mesothelioma (MPM) patients lacking *PTEN* expression has been observed [78, 84–85]. mTOR signaling pathways has been associated with shortened survival in patients with malignant peritoneal mesothelioma [86]. ROS are both upstream and downstream of mTOR. Activation of the PI3K/TOR pathway increases production of ROS, whereas inhibition of mTOR decreases ROS levels [87]. ROS have been reported to play a role in cell survival by mediating cellular signal transduction pathways. These signaling pathways are involved in the transmission of inter- or intracellular information, and are critical for supporting tumor cell survival and establishing cell fate. The reduced nicotinammide adenine dinucleotide phosphate oxidase (NOX) family of enzymes, one of the potential sources of ROS production, has been reported to promote tumor cell survival and growth [88]. In mesothelioma cells, higher superoxide production and NOX4 expression were observed as compared to mesothelium [89]. Consequently, ROS potentiate the survival pathways in MM.

### ROS and mesothelioma cell proliferation

Uncontrolled proliferation is a hallmark of cancer cells. ROS and RNS inhibit or promote cell proliferation by modulating the cell signaling pathways that dictate decisions between cell survival, proliferation, and death. In the growth factor-dependent pathways that regulate mitogenesis, numerous positive and negative effectors of signaling are influenced by physiological fluctuations of oxidants, including receptor tyrosine kinases, small GTPases, mitogen-activated protein kinases (MAPK), protein phosphatases, and transcription factors. The same mitogenic pathways that are sensitive to oxidant levels, also directly regulate the expression of cyclin D1, a labile factor required for progression through the G1 phase on the cell cycle. Because the transition from G0 to G1 is the only phase of the cell cycle that is regulated by redox-dependent signaling pathways, expression of cyclin D1 represents a primary regulatory node for the dose-dependent effects of oxidants on the induction of cell growth [90].

Induction of MAPK signaling pathways occurs in response to exposure to asbestos and appears to be related to ROS. The MAPK cascade is characterized by a sequential series of phosphorylation events catalyzed by ERK, c-jun NH2-terminal kinases (JNK) or stress-activated protein kinases (SAPK), and p38 [91], that promote cellular responses, such as proliferation, apoptosis or inflammation [92]. Asbestos fibers selectively induce ERK phosphorylation and activity in mesothelial cells, leading to apoptosis and/or cell proliferation [71, 93–94].

MAPK signaling cascades phosphorylate and activate transcription factors such as activator protein 1 (AP-1) [95]. AP-1 is a family of transcription factors comprised of homo- and heterodimers of the Jun and Fos early response proto-oncogenes. It is a redox-sensitive transcription factor classically associated with the development of cell proliferation and tumor promotion [96]. Asbestos is able to induce AP-1 activation through the activation of MAPK family members, ERK1 and ERK2, in *in vitro* experiments [97]. Asbestos also induces a dose-dependent activation of NF-kB, a redox sensitive transcription factor [98, 99]. NF-kB triggers the activation of a number of genes involved in cell proliferation and apoptosis, including cytokines, growth factors, and adhesion molecules as well as proto-oncogenes such as c-myc [100]. Detailed studies to determine how asbestos regulates the transcription factors NF-kB and AP-1, led to the evidence that asbestos-generated ROS could be a possible mechanism [101]. Kinases, such as protein kinase C (PKC), can also be activated by H_2_O_2_ and redox cycling quinones [102]. PKC is involved in asbestos-induced proto-oncogene (Fos/Jun) expression in mesothelial cells, and the down-regulation or inhibition of PKC prevents asbestos-induced proto-oncogene expression [103]. Asbestos fibers, either by elaboration of oxidants or interaction with the cell membrane induce mitogenesis and cell proliferation.

### ROS and mesothelioma cell invasion

Human pleural malignant mesothelioma is characterized by aggressive local spreading into the pleura and the surrounding tissues, but it has a low rate of distant metastasis [104]. Malignant tumor invasion of normal tissue involves three independent processes: degradation of the extracellular matrix, cell migration, and proliferation. Matrix metalloproteinases (MMPs) are the main groups of enzymes involved in the proteolysis of extracellular matrix proteins, such as collagen, proteoglycans, elastin, laminin, and fibronectin. MMP-2 and MMP-9, key enzymes in the degradation of type IV collagen (the major component of the basement membrane) are abundantly expressed in various malignant tumors and contribute to invasion and metastasis [105].

MMPs have been shown to be elevated in mesothelioma and are known to increase the invasive potential in mesothelioma cells [106]. MMP-2 and MMP-9 expression was reported as a characteristic for pleural malignant mesothelioma, and particularly MMP-2 was suggested as a predictive marker for poor prognosis [107, 108]. Exposure of cells to H_2_O_2_ increases MMP-2 activation via a receptor tyrosine kinases/PI3-kinase/NF-κB activation. Oxidative stress may also modulate MMP expression by activation of Ras, or direct activation of the MAPK family members ERK1/2, p38, and JNK, or inactivation of phosphatases that regulate these proteins [109]. In addition, ROS have been implicated in MMP gene expression. Both hydrogen peroxide and nitric oxide donors, as well as the increased expression of iNOS, stimulate the expression of several MMPs [110].

Several studies have reported the involvement of chemokines and chemokine receptors in the invasion and metastasis of various types of tumors. The metastatic potential of chemokines is attributed to their ability to induce the expression of MMPs, which facilitate tumor invasion [111]. Chemokine synthesis is induced in various cells by inflammatory stimuli. Pleural mesothelial cells were observed to produce chemokines on stimulation by inflammatory mediators, asbestos, and ROS [25]. The chemokine CXCL12 (stromal derived factor 1) binding to its receptor CXCR4 may mediate cell adhesion, migration, and proliferation in tumor cells. CXCL12 and CXCR4 were overexpressed in mesothelioma. CXCR4 was found in almost all mesotheliomas (97%) and CXCL12 in 78%; another receptor, CXCR7, was only weakly expressed [112].

H_2_O_2_ also influences cell-cell interaction [105]. The CD44 hyaluronic acid receptor, a cell-surface glycoprotein involved in cell-cell interactions, is highly expressed in human mesotheliomas and mediates the association with hyaluronan, a major component of pleural fluid [113]. Mesothelioma cell lines with the highest amount of CD44 receptor show increased proliferation and migration when stimulated with low molecular weight hyaluronic acid. Furthermore, the use of a monoclonal antibody against CD44 inhibits proliferation by 12–40% and migration by 10–35% in mesothelioma cell lines [114]. Exemestane, a drug that acts by reduction of CD44, inhibits proliferation and migration in mesothelioma cells [115]. CD44 is influenced by ROS, measured by hydrogen peroxide treatments [116]. Microarray (Affymetrix) data comparing rat pleural mesothelial cells (with and without exposure to crocidolite asbestos) and rat mesotheliomas indicate that CD44 was increased in mesotheliomas and in mesothelial cells after acute exposure to asbestos [117].

### ROS and mesothelioma cell angiogenesis

Angiogenesis is required for tumors to grow beyond a certain size and to metastasize. The development of a clinically observable tumor requires the neoformation of a vascular network sufficient to sustain tumor growth [118]. A number of cellular stress factors, including hypoxia, nutrient deprivation, and ROS are important stimuli of angiogenic signalling. Asbestos induced angiogenesis surrounding 20–30% of the lesions after six weekly iniectjons in mice [119]. To develop a stable blood supply for tumor growth, many cells in the tumor microenvironment, including tumor epithelial cells, stromal cells, and immune cells, secrete various proangiogenic factors that stimulate endothelial cell recruitment, proliferation, migration, and tubule formation [120]. Tight regulation of the dynamic equilibrium between proangiogenic and antiangiogenic factors is critical to health, as an imbalance in either direction contributes to a wide range of pathological conditions from atherosclerosis to cancer [121].

A large number of proangiogenic factors and their cognate receptors have been identified, including among the others, Vascular Endothelial Growth Factor (VEGF), Placenta Growth Factor (PlGF), Platelet-Derived Growth Factor (PDGF) and acidic and basic Fibroblast Growth Factors (FGF-1 and -2, respectively). Central to the physiological and pathological regulation of angiogenesis is the VEGF system, its ligands and receptors (VEGFRs). VEGF is the most potent direct-acting angiogenic protein known. It elicits a pronounced angiogenic response in a variety of *in vivo* models [122]. VEGF has been identified as an important mediator of angiogenesis in malignant mesothelioma. The significant higher levels of VEGF found in the pleural exudates of patients with malignant mesothelioma compared with patients with non malignant pleural disease and the detection of a significant inverse correlation between serum VEGF and malignant mesothelioma patient survival confirm VEGF as an important mediator of angiogenesis [123].

### ROS and mesothelioma cell death

There are three major ways by which a cancer cell can die: apoptosis, necrosis, and autophagy [124–125]. ROS can induce cell death by apoptosis, necrosis, and autophagy [125–127].

### ROS and apoptosis

Resistance to apoptosis may be important both for the initial development and for the subsequent survival of tumors. An initial resistance to apoptosis may be necessary to allow the amplification of an abnormal cell population [128]. Apoptosis is a tightly controlled form of cell death and can be initiated by death receptors (extrinsic pathway) or by mitochondria (intrinsic pathway). Both extrinsic and intrinsic pathways of apoptosis depend on ROS [126]. The extrinsic pathway of apoptosis is mediated by death receptors in which ligand-receptor binding initiates protein-protein interactions at cell membranes that, in turn, activate the initiator caspases. Major known receptors include Fas (also called CD95 or APO-1), TNF receptor 1 (TNFR1) and TNF-related apoptosis-inducing ligand (TRAIL) receptor 1 (TRAIL-R1; also called DR4) and TRAIL receptor 2 (TRAIL-R2; also called DR5) [129, 130]. ROS are required for the ubiquitination and subsequent degradation of the FLICE inhibitory protein to further enhance Fas activation [131]. The intrinsic or mitochondrial apoptotic pathway is characterized by the opening of the permeability transition pore complex on the mitochondrial membrane, which results in cytochrome c release, apoptosome formation, and caspase activation. Opposing effects of pro-apoptotic and anti-apoptotic Bcl-2 family proteins are required to open the permeability transition pore. In this context, ROS open the pore by both activating pore-destabilizing proteins (Bcl-2-associated X protein, Bcl-2 homologous antagonist/killer) and inhibiting pore-stabilizing proteins (Bcl-2 and Bcl-xL) [132]. MM cell lines have been shown to be highly resistant to oxidant (asbestos and ROS) and non oxidant-induced apoptosis, and this resistance is not explained by Bcl-2 [133]. The expression of apoptosis-regulating proteins (Bcl-2/Bax and Fas/FasL) and their prognostic significance in asbestos-induced MPM were analyzed in patients with MPM. The findings indicate that Bcl-2 may not be involved in the tumorigenesis of MPM [134]. One explanation for why MM cell lines are more resistant than non-transformed cells is that human MM cell lines have increased Mn-SOD and catalase mRNA levels and activity that render cells more resistant to the cytotoxic effects of an oxidant stress [135]. Notably, cells transfected with Mn-SOD are resistant to apoptosis caused by TNF-α, H_2_O_2_, and irradiation [136]. These data suggest that asbestos-induced ROS play a critical role in mediating apoptosis and that increased activity of antioxidant defences, especially Mn-SOD and catalase, accounts in part for the resistance of MM cells to apoptosis. Poly-ADP-ribose polymerase (PARP), a nuclear enzyme, is activated by DNA strand breaks. Prolonged PARP activation can deplete cellular NAD and ATP levels, and thereby augment cell death [137]. PARP may be particularly important since the onset of ROS-induced apoptosis is closely associated with the production of PARP-cleavage products, and reduced PARP activity may impair normal cellular DNA repair mechanisms [138]. PARP activation is also implicated in mediating asbestos-induced mesothelial cell apoptosis, since the PARP inhibitor 3-aminobenzamide (3-ABA) is protective. All this, together with other evidence not discussed here, proves that apoptosis induction in mesothelial cells via ROS represents a mechanism by which mesothelial cells with asbestos-induced DNA damage are deleted. If so, escape from the normal apoptotic pathway may be one important step in the multistep process leading to the development of asbestos-induced neoplasia [139].

### ROS and necrosis

Necrotic cell death has been proposed to involve ROS accumulation [140]. The necrotic pathways, as well as apoptosis, ensure that cells with irreparable damage are eliminated. Necrosis is a form of cell injury that results in the premature death of cells in living tissue. Necrosis is caused by factors external to the cell or tissue, such as infections, toxins, or traumas that result in the unregulated digestion of cell components. Dying cells release the products of cell death into the extracellular space, leading to an anti-inflammatory response in the surrounding tissue [141]. Asbestos causes mesothelial necrotic cell death and promotes an inflammatory response. Macrophages and mesothelial cells release ROS, such as H_2_O_2_ and secrete TNF-α, amplifying the inflammatory process. Moreover, ROS cause DNA damage and aneuploidy. TNF-α activates NF-κB, a survival pathway that allows some mesothelial cells undergoing asbestos-induced DNA damage to survive, thereby creating a pool of aneuploid mesothelial cells with the potential to develop into cancer cells. This mechanistic rationale links asbestos-induced mesothelial cell death to the chronic inflammatory reaction that is associated with asbestos carcinogenesis [142].

### ROS and autophagy

Autophagy, a process by which eukaryotic cells degrade and recycle macromolecules and organelles, has an important role in the cellular response to oxidative stress. Autophagy is triggered and regulated by ROS, as revealed by several recent studies [143, 144]. The outcomes of autophagy vary from survival, by promoting the removal of pathogens, damaged organelles, and proteins, to programmed cell death. Thus, ROS may act as signaling molecules in autophagic cell death, despite they may also act as signaling molecules in survival-prone autophagy [144]. Chrysotile asbestos-induced autophagy is mediated by ROS in A549 human lung epithelial cells [145]; while it does not appear to play a role in mesothelial cells [142].

### ROS and mesothelioma therapy

MM remains a rare and lethal disease, difficulties in MM diagnosis and staging, especially of early disease, have thwarted the development of a universally accepted therapeutic approach. There is no definite standard of care, and only a minority of patients are eligible for any potentially curative therapy. Single modality therapies (surgery, radiotherapy, chemotherapy) have generally failed to significantly prolong patient survival [146]. Surgical resection is the only curative treatment of MPM while other treatments minimally improve the response rate and overall survival. The majority of patients diagnosed with MPM are unable to undergo surgical resection because of advanced disease at time of presentation. The only first line chemotherapy regimen approved by the FDA for these patients is cisplatin plus an antifolate, such as pemetrexed or raltitrexed, which improves overall survival from 9 months to 12 months [147, 148]. Radiotherapy may be used for palliative care; however, there is no evidence for routine use of radiation as primary therapy for MPM [149]. For patients with surgically resectable MPM, surgical options include extrapleural pneumonectomy or pleurectomy with decortication which may be combined with intracavitary chemotherapy at the time of resection or systemic chemotherapy and radiotherapy [150, 151]. Unfortunately, only a small percentage of MPM patients will qualify for multimodality treatment and despite this aggressive therapy, MM recurrence is frequent. Diffuse malignant peritoneal mesothelioma (DMPM) makes up 15–20% of MM diagnoses and like MPM, is typically diagnosed at late stage [152]. DMPM is ultimately fatal although advances have been made in therapeutic strategies for surgically resectable diseases. The combination of cytoreductive surgery and hyperthermic intraperitoneal chemotherapy with cisplatin plus doxorubicin or mitomycin-C has been shown to improve overall survival [152]. Patients with inoperable DMPM may undergo systemic chemotherapy with cisplatin and pemetrexed and/or palliation surgery.

### ROS-generating drugs and radiotherapy

**Figure 4:**
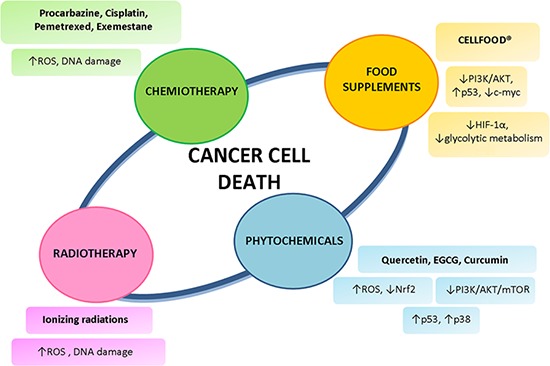
Non-surgical strategies for mesothelioma treatment Different types of mesothelioma treatment employ not only the standard protocols of chemiotherapy and radiotherapy, but also rely on phytochemicals and food supplements to induce cancer cell death.

ROS production is a mechanism shared by all non-surgical therapeutic approaches for cancers, including chemotherapy and radiotherapy, due to their implication in triggering cell death; therefore, ROS are also used to kill cancer cells [140]. Based on either side, a number of drugs, agents and approaches have been developed or are under development (Figure 4).

One of the first ROS-generating drugs to be developed was procarbazine. It is oxidised readily in an oxic environment to its azo derivative, generating ROS [154]. A synergistic effect in DNA degradation when procarbazine was combined with radiation was reported. In a study involving 35 patients with mesothelioma treated with either radiation alone or radiation plus procarbazine, none of the 9 patients treated with radiotherapy alone responded, whereas 14 of the 26 patients treated with procarbazine and radiation responded subjectively or objectively. Moreover, the tumour shrinkage in the combined-modality arm was only observed within the radiation field, whereas procarbazine had no effect on the disease outside of the radiation port [155]. Therefore, ROS-generating drugs can be used as a cancer treatment, and perhaps to enhance the antitumour effect of radiation therapy. Radiation therapy involves the administration of ionizing radiation. When cells are ionized, free radicals and ROS are formed. These agents, due to their high reactivity, are likely to attack the covalent bonds of DNA and other cells they encounter, and these reactions typically occur in chains. Enough injury in the cell will result in apoptosis, or programmed cell death. At the same time, if enough DNA is damaged, the cells will be unable to replicate. Thus, when the radiation targets the tumor cells, the affected cells will die or be unable to proliferate, effectively reducing or eliminating the cancer [156]. Though radiation therapy has often resulted in remission of cancer, recurrence is fairly common. Recent research has found that this might be due to cancer stem cells producing higher levels of antioxidant proteins than other cancer cells. The antioxidants capture and disarm ROS before they cause too much damage. Thus, even though it seems that most of the cancer cells have been killed, some cancer stem cells remain and proliferate over time due to the antioxidant defense against ionizing radiation [157]. As cancer cells have elevated ROS generation and are under increased intrinsic oxidative stress, it is conceivable that these malignant cells would be more dependent on antioxidants for cell survival and, therefore, more vulnerable to further oxidative insults induced by ROS-generating agents or by compounds that abrogate the key antioxidant systems in cells. As such, manipulating ROS levels by redox modulation seems to be a feasible way to selectively kill cancer cells with less toxicity to normal cells [158]. The idea of inducing preferential cancer cell death by a ROS-mediated mechanism based on the different redox states in normal and malignant cells was proposed a decade ago [159, 160], but its feasibility has only recently gained momentum [161–163]. Cisplatin is one of the most effective and widely used anticancer agents for the treatment of several types of tumors. The cytotoxic effect of cisplatin is thought to be mediated primarily by the generation of nuclear DNA adducts, which, if not repaired, cause cell death as a consequence of DNA replication and transcription blockage [164, 165]. Cisplatin exposure induces a mitochondria-dependent ROS response that significantly contributes to cell killing by enhancing the cytotoxic effect exerted through the formation of DNA damage [166]. In MM cells, cisplatin treatment produced higher ROS levels in MSTO-211H than in NCI-H2452 cells, corresponding to a greater sensitivity of MSTO-211H to the drug. ROS elevation by cisplatin is markedly decreased in the presence of N-Acetyl Cysteine (a ROS scavenger), suggesting that in MM the production of ROS is implicated in the action of cisplatin [167]. Pemetrexed is a first line therapy against mesothelioma. Pemetrexed targets the folate-dependent enzymes thymidylate synthetase (TS), dihydrofolate reductase (DHFR), and glycinamide ribonucleotide formyltransferase (GARFT), all of which are involved in the de novo biosynthesis of purines and pyrimidines, thereby inducing an imbalance in the nucleotide pool and consequent DNA damage [168]. Pemetrexed induces caspase-dependent and -independent apoptosis in human melanoma cells through intracellular ROS accumulation, which in turn promotes DNA damage [169]. In MM cell lines, pemetrexed induced ROS production and caspase-dependent apoptosis [170]. To the best of our knowledge, no previous study has investigated on ROS production in mesotlelioma cells upon pemetrexed and cisplatin combination treatment. Some authors administered a combination of cisplatin, pemetrexed, and valproate (histone deacetylase inhibitor) to three different histological types of mesothelioma (epithelioid, sarcomatoid, and biphasic) cell lines, and found that the number of apoptotic cells increased relative to the first line chemotherapy regimen results (cisplatin + pemetrexed). They also showed that induction of apoptosis resulted from the production of ROS [171]. We recently demonstrated that exemestane, effective in the treatment of MM in *in vivo* and *in vitro* MM experimental models, also acts through ROS production [115, 172, 173].

### ROS and natural compounds in mesothelioma treatment

A large number of dietary phytochemicals has been demonstrated to exhibit anticancer activities by interfering with multiple signaling pathways, resulting in inhibiting survival proteins or activating proapoptotic mediators [174] (Figure 4). In addition, a number of dietary phytochemicals exhibit synergistic effects with conventional chemotherapy and radiotherapy. Of those, resveratrol, a naturally occurring polyphenolic phytoalexin with antioxidant and anti-inflammatory effects, has been identified as an effective candidate for overcoming chemoresistance in tumor cells. Many mechanisms of action have been postulated in order to explain the antiproliferative activity of resveratrol including the generation of ROS [175]. In particular, a synergistic anti-proliferative effect occurred in MM cells (MSTO-211H) when resveratrol was combined with the chemotherapeutic drug clofarabine. Such a synergism included simultaneous targeting of multiple biological pathways involving activation of p53 [176], reduction of Nrf2 activity [177], and suppression of Sp1 and PI3-kinase/Akt survival proteins [178]. However, despite the large number of preclinical studies dealing with different aspects of the biological effects of resveratrol, its translation to clinics is far from reality due to a variety of challenges [179]. Quercetin is an important dietary flavonoid with diverse biological activities, including antioxidant, anti-inflammatory and antitumor properties. Collectively the proapoptotic effects of Quercetin may result from multiple pathways and ROS generation [180]. Experiments on SPC212 and SPC111 mesothelioma cell lines showed that quercetin significantly reduced cell proliferation, altered the cell cycle distribution, and increased the level of Caspase-3 and -9 [181]. Interestingly, the combination of quercetin with cisplatin was found more effective when compared with individual treatment of agents. Other experiments on MSTO-211H mesothelioma cells revealed that quercetin interacted with the transcription factor Sp1 and significantly suppressed its expression at the protein and mRNA levels. Furthermore, quercetin modulated the levels of Sp1 regulatory genes, such as cyclin D1, myeloid cell leukemia (Mcl)-1 and surviving [182]. Epigallocatechin-3-gallate (EGCG), a natural polyphenol component of green tea, has been extensively studied for its anticarcinogenic effect in a wide variety of cancer cells. Even though EGCG is generally known as an antioxidant, mounting evidence points a role in enhancing ROS release, which in turn inhibits tumor growth [183]. In line with these findings, EGCG was more cytotoxic for MM cells than for normal mesothelial cells, through a mechanism of action based on extracellular H_2_O_2_ production, Ca^2+^ homeostasis loss, and intracellular ROS increase [184]. At the same time, a negative modulation of mitochondrial oxidative phosphorylation by EGCG leading to growth arrest and apoptosis has been evidenced in MM cells [185]. A series of *in vitro* tests on MM cells have revealed a synergistic cytotoxicity of EGCG in combination with the conventional tumor drug gemcitabine and with ascorbate (mixture called AND, for Active Nutrients/Drug) through cell cycle deregulation and apoptosis induction [186, 187]. Interestingly, *in vivo* experiments on a xenograft mouse model for MM, obtained by REN cells injection in immunocompromised mice, showed that AND strongly reduced the size of primary tumor as well as the number and size of metastases [188]. At the cellular level, there was a shift from cell proliferation to apoptosis in the outermost layer of tumor mass, concomitantly with the inactivation of kinases involved in cell growth. Hence, the AND combination has been proposed as a new treatment for MM. Curcumin is a naturally occurring polyphenol in the spice turmeric, which comes from the rhizomes of the herb *Curcuma longa*. Prior research has identified a broad range of anticarcinogenic potential of curcumin in various cancer types through its antioxidant, anti-inflammation, antiproliferative, antiangiogenic, proapoptotic, and enhancing chemoradiation properties [189]. In human (H2373, H2452, H2461, and H226) and murine (AB12) MM cells, curcumin inhibited cell growth in a dose- and time-dependent manner, while pre-treatment of MM cells with curcumin enhanced cisplatin efficacy [190]. In the same study, curcumin activated the stress-activated p38 kinase, caspases-9 and -3, caused elevated levels of proapoptotic proteins Bax, stimulated PARP cleavage, and apoptosis. In addition, oral administration of curcumin inhibited growth of murine MM cell-derived tumors *in vivo* in part by stimulating apoptosis. However, in the ACC-MESO-1 human MPM cell line, curcumin administration dose-dependently reduced cell viability by inducing autophagy and not apoptosis [191]. Recently, it has been demonstrated that curcumin induces cytotoxic effects on malignant mesothelioma cells (HMESO) through pyroptosis in a process involving ROS production [192]. In addition, curcumin had anti-inflammatory effects by blocking cytokine processing of IL-1β and IL-18 and genes involved in the NF-kB pathway. Overall, these results provide evidence that curcumin warrants further investigation as a therapeutic agent in MM, although future studies must include improved curcumin analogs or enhanced modes of delivery to overcome curcumin's most challenging feature, which is limited bioavailability. Other than protecting biomolecules and cells from oxidative damage [193], evidence has been recently supplied for an anti-cancer activity of Cellfood™ (CF) [194, 195]. Apoptotic death was observed in CF-treated MSTO-211H. Interestingly, preliminary data indicate that CF, at the doses used to kill MM cells, induces an increase of ROS.

## CONCLUSION

This review implicates the role of ROS in MM pathogenesis. ROS are usually increased in MM cells due to oncogene activation, and are involved in initiation and progression of MM. Ironically, ROS production is a mechanism shared by all non-surgical therapeutic approaches for cancers, including chemotherapy and radiotherapy, due to their implication in triggering cell death, therefore ROS are also used to kill cancer cells. Because of the double-edged sword property of ROS in determining cell fate, both pro- or anti-oxidant therapies have been proposed for cancer treatment. Based on either side, a number of drugs, agents and approaches have been developed or are under development, some of which have shown clinical promise. This review summarizes the role of ROS in various phases of tumorigenesis and the current understanding on ROS-manipulation strategies in MM treatment.
